# The Relationship between Dark Triad Personality Traits and Sexting Behaviors among Adolescents and Young Adults across 11 Countries

**DOI:** 10.3390/ijerph18052526

**Published:** 2021-03-04

**Authors:** Mara Morelli, Flavio Urbini, Dora Bianchi, Roberto Baiocco, Elena Cattelino, Fiorenzo Laghi, Piotr Sorokowski, Michal Misiak, Martyna Dziekan, Heather Hudson, Alexandra Marshall, Thanh Truc T. Nguyen, Lauren Mark, Kamil Kopecky, René Szotkowski, Ezgi Toplu Demirtaş, Joris Van Ouytsel, Koen Ponnet, Michel Walrave, Tingshao Zhu, Ya Chen, Nan Zhao, Xiaoqian Liu, Alexander Voiskounsky, Nataliya Bogacheva, Maria Ioannou, John Synnott, Kalliopi Tzani-Pepelasi, Vimala Balakrishnan, Moses Okumu, Eusebius Small, Silviya Pavlova Nikolova, Michelle Drouin, Antonio Chirumbolo

**Affiliations:** 1 Department of Dynamic and Clinical Psychology and Health Studies, Sapienza University of Rome, 00185 Rome, Italy; 2 Department of Psychology, Sapienza University of Rome, 00185 Rome, Italy; flavio.urbini@uniroma1.it (F.U.); antonio.chirumbolo@uniroma1.it (A.C.); 3 Department of Developmental and Social Psychology, Sapienza University of Rome, 00185 Rome, Italy; dora.bianchi@uniroma1.it (D.B.); roberto.baiocco@uniroma1.it (R.B.); fiorenzo.laghi@uniroma1.it (F.L.); 4 Department of Human and Social Sciences, University of Valle D’Aosta, 11100 Aosta, Italy; e.cattelino@univda.it; 5 Institute of Psychology, University of Wroclaw, 50-527 Wrocław, Poland; sorokowskipiotr@yahoo.co.uk (P.S.); michalmis44@gmail.com (M.M.); 6 Department of Psychology and Cognitive Science, Adam Mickiewicz University, 60-658 Poznań, Poland; mdzieka1@gmail.com; 7 Department of Health Sciences, University of Central Arkansas, Conway, AR 72035, USA; heatherh@uca.edu; 8 College of Public Health, University of Arkansas for Medical Sciences, Little Rock, AR 72205, USA; smarshall@uams.edu; 9 College of Education, University of Hawaii at Manoa, Honolulu, HI 96822, USA; nguyen@hawaii.edu (T.T.T.N.); lmark@hawaii.edu (L.M.); 10 Centre for Prevention of Risky Virtual Communication, Palacky University Olomouc, 77900 Olomouc, Czech Republic; kopeckyk@seznam.cz (K.K.); rene.szotkowski@upol.cz (R.S.); 11 Psychological Counseling and Guidance, MEF University, Istanbul 34396, Turkey; ezgitoplu@hotmail.com; 12 Department of Communication Studies, University of Antwerp, 2000 Antwerp, Belgium; joris.vanouytsel@uantwerpen.be (J.V.O.); michel.walrave@uantwerpen.be (M.W.); 13 Hugh Downs School of Human Communication, Arizona State University, Tempe, AZ 85281, USA; 14 Department of Communication Sciences, imec-mict-Ghent University, 9000 Ghent, Belgium; Koen.Ponnet@ugent.be; 15 Chinese Academy of Sciences, Beijing 100864, China; tszhu@psych.ac.cn (T.Z.); chenya19930415@163.com (Y.C.); zhaonan@psych.ac.cn (N.Z.); liuxiaoqian@psych.ac.cn (X.L.); 16 Department of General Psychology, Lomonosow Moscow State University, 101000 Moscow, Russia; vae-msu@mail.ru; 17 Department of Pedagogy and Medical Psychology, Sechenov University, 101000 Moscow, Russia; bogacheva.nataly@gmail.com; 18 Department of Psychology, University of Huddersfield, Huddersfield HD1 3DH, UK; m.ioannou@hud.ac.uk (M.I.); j.p.synnott@hud.ac.uk (J.S.); k.tzanipepelasi@hud.ac.uk (K.T.-P.); 19 Department of Information System, University of Malaya, Kuala Lumpur 50603, Malaysia; vimala.balakrishnan@um.edu.my; 20 School of Social Work, University of North Carolina, Chapel Hill, NC 27599, USA; moses.okumu@mavs.uta.edu; 21 School of Social Work, University of Texas at Arlington, Arlington, TX 76019, USA; esmall@uta.edu; 22 Department of Social Medicine and Healthcare Organization, Medical University-Varna, 9000 Varna, Bulgaria; silviya.p.nikolova@mu-varna.bg; 23 Department of Psychology, Purdue University Fort Wayne, Fort Wayne, IN 46805, USA; drouinm@pfw.edu

**Keywords:** sexting, dark triad traits, personality, cross-country investigation, adolescents, young adults

## Abstract

Background: Sexting is an increasingly common phenomenon among adolescents and young adults. Some studies have investigated the role of personality traits in different sexting behaviors within mainstream personality taxonomies like Big Five and HEXACO. However, very few studies have investigated the role of maladaptive personality factors in sexting. Therefore, the present study investigated the relationship between Dark Triad Personality Traits and experimental (i.e., sharing own sexts), risky (i.e., sexting under substance use and with strangers), and aggravated sexting (i.e., non-consensual sexting and sexting under pressure) across 11 countries. Methods: An online survey was completed by 6093 participants (Mage = 20.35; SDage = 3.63) from 11 different countries which covered four continents (Europe, Asia, Africa, and America). Participants completed the Sexting Behaviors Questionnaire and the 12-item Dark Triad Dirty Dozen scale. Results: Hierarchical regression analyses showed that sharing own sexts was positively predicted by Machiavellianism and Narcissism. Both risky and aggravated sexting were positively predicted by Machiavellianism and Psychopathy. Conclusions: The present study provided empirical evidence that different sexting behaviors were predicted by Dark Triad Personality Traits, showing a relevant role of Machiavellianism in all kinds of investigated sexting behaviors. Research, clinical, and education implications for prevention programs are discussed.

## 1. Introduction

The emergence and spread in the use of the Internet and the smartphones in interpersonal communication, including sexual communication, has increased greatly over the past 20 years. Sexting is a sexual communication characterized by the sending or receiving of sexts, that is text messages, photos and/or videos with sexually explicit or provocative content, via technological devices. Although sexting occurs at all ages, it is especially prevalent among adolescents and young adults [1,2,3,4].

Over the past decade, research on sexting has been focused on several areas of interest. However, although many studies have considered sexting from different research perspectives, the picture that has emerged from these investigations is not uniform due to different definitions (e.g., text-based sexting versus visual forms of sexting), evaluation methods [5,6,7], and research perspective applied. A review of the literature suggests that a great deal of theoretical and empirical work is still required to capture the effects and nature of sexting [2,8,9]. A growing body of literature investigated the relationship between sexting and different variables. Some studies have found that sexting behaviors tend to grow with increasing age, specifically from adolescence to young adulthood [1,8]. In other cases, studies have not yet established a conclusive link between some socio-demographic variables and sexting behaviors, such as age [10], gender [1], and sexual orientation (e.g., [11,12,13,14,15,16]). Other findings suggest that young adults had more positive expectations and attitudes toward sexting with a committed partner, rather than with a casual dating partner [17,18].

Generally speaking, a rough distinction can be drawn regarding the positive (i.e., experimental sexting) and negative aspects of sexting (i.e., aggravated or risky sexting) [19]. In the following paragraphs we will discuss the differences between (1) experimental sexting, (2) aggravated sexting, and (3) risky sexting.

An increasing amount of scholars consider sexting as a normal, even healthy aspect of sexual expression, and part of the repertoire of interpersonal sexual communication relationships (e.g., [15,20,21]). This kind of sexting, named experimental sexting, refers to consensual exchange of sexual content for addressing young peoples’ developmental tasks and needs, such as exploring their sexuality and identity [22,23]. Wolak, Finkelhor, and Mitchell [19] were the first to talk about the concept of experimental sexting, identifying sexting as a new sexual normative behavior related to sexual experimentation that is typical of adolescence and young adulthood, such as sharing own photos for receiving feedback about the adequacy of one’s own body image or for sexual experimentation [24,25]. Indeed, for some adolescents, sexting can also function as a first step toward offline sexual contact [26]. In line with this positive perspective, sexting, especially with a committed partner, has been associated with fun, intimacy, and passion [27,28], facilitates communication among sexual minorities [29], is used for body image reinforcement, and increases self-esteem [30]. In a recent literature review on sexting, it was concluded that sexting behaviors are becoming more prevalent among young adults within dating and romantic relationships [31].

Whereas experimental sexting can generally be perceived as positive, sexting may also have a negative side [31]. In fact, recent reviews and meta-analyses of the literature identified two further kind of sexting: aggravated sexting and risky sexting [1,2,19,32]. Aggravated sexting can encompass experiences of unwanted sexting, unauthorized dissemination of sexts, and coercion [31]. It is these latter problematic forms of sexting that can have significant negative consequences on the victims, particularly when sexting involves harmful intentions [19]. This kind of sexting, referred to as aggravated sexting, refers to harmful behaviors such as publicly sharing sexts of someone without their consent [1,14,24] or coercive sexting under threats or pressure by a partner or friends [27,33,34]. Thus aggravated sexting can include dimensions of perpetration (i.e., non-consensual sexting, that is sharing sexts of someone else without their consent) as well as victimization (i.e., being pressured to sext).

A meta-analysis [23] found inconsistent findings regarding the relationship between sexting and risky sexual behaviors: some studies found that sexting was associated with high-risk sexual practices, such as having a higher average of lifetime sexual partners (e.g., [35,36]) or having had sex without protection (e.g., [37]). Other studies, however, have found no association or no longitudinal association between sexting and risky sexual behaviors (e.g., [26,38]). Considering these results, the authors of the meta-analysis [23] suggested that sexting may not be a particularly good indicator of offline risky sexual behaviors. Moreover, the majority of these studies used cross-sectional data [39]; therefore, it is not advisable to draw causal inferences regarding sexting as an antecedent of risky sex. It is possible that the relationship between sexting and risky sexual behaviors can be explained by an underlying variable, such as pubertal timing or participating in risky behaviors.

Conversely, another recent systematic review of the literature and meta-analysis found that there is a strong association between sexting and different kind of risky and sexual behaviors [32]. Some studies found a relationship between sexting and substance use [40], depression [40], feelings of sadness or hopelessness, and attempted suicide [35], lower levels of psychological well-being [41], and less confidence in social skills [42]. Regarding potentially unhealthy behaviors such as drinking, smoking, or severe substance use, results found that sexting behaviors are significantly related to the use of different recreational drugs, including alcohol, marijuana, ecstasy, and cocaine (e.g., [43]). However, Temple and colleagues [44] argued that the relationship between teen sexting and substance use is spurious, possibly explained by some underlying mechanism related to poor parental monitoring or socializing with delinquent peers. The association between sexting and other risky behaviors may also depend on the relationship context, as one study found that associations between sexting and risk behaviors were more prevalent outside of a romantic relationship than within a romantic relationship [45]. Therefore, in line with these studies [40,41,42,43], it is important to distinguish a third particular kind of sexting, that we called risky sexting. With risky sexting, we refer to sexting in conjunction with other risk behaviors, such as sexting under the influence of alcohol or drugs or sexting with strangers or people known only online. In risky sexting there is no coercive dynamic of violence in which the sexter is a victim or perpetrator of violence (as is the case with aggravated sexting) but a co-occurrence of multiple risky behaviors associated with sexting that can be related to an underlying common etiological pattern, such as common psychopathological personality traits, as suggested by Morelli et al. [40].

Thus far, the literature suggests that sexting can represent either normal and risky sexual behaviors, or both; however, this might be dependent on the surrounding circumstances and the individual traits of the individual engaged in sexting. Hence, personality traits may offer an important contribution in our understanding of different kinds of sexting behaviors.

With respect to differences in sexting by country, findings showed some differences probably due to cultural values within a society [46]. This assumption is grounded on the evidence that contextual factors, such as cultural values, may influence adolescents’ and young adults’ online behavior. In this regard, for example, European countries located in north-west, south, and east showed some inhomogeneity toward sexual permissive attitudes [46].

Regarding experimental and aggravated sexting, the recent meta-analysis by Mori and colleagues [2], based on fifty studies among young adults in different countries, revealed different prevalence rates. Twenty-seven of these fifty studies reported data gathered in different continents (i.e., Europe, Africa, Australia, North and South America). Findings showed that over a third of young adults reported that they have been involved in experimental sexting, whereas the 15% of the total sample reported to be involved in aggravated sexting. In particular, the European countries, such as Czech Republic (N = 1), Croatia (N = 2), and Spain (N = 1) are characterized by the prevalence of aggravated sexting than other countries where experimental sexting is more prevalent, such as America (N = 11), Australia (N = 1), Africa (N = 1), Canada (N = 1). Interestingly, young adults in other countries are involved just in sexually explicit text messages, such as Mexico (N = 1) and Nigeria (N = 1). Less is known about experimental and aggravated sexting among Indian and Chinese young adults included in the study. A study conducted on young adults in Hong Kong found that only 18% of participants reported to have been involved in experimental sexting [47]. This could probably due to the lower level of sexual permissiveness in Chinese than in Western culture [48].

Conversely, comparing the results by Mori et al. [2] with the results of the systematic review and meta-analysis of Madigan et al. [1] on consensual and aggravated sexting among adolescents, results showed that only 14.8% of adolescents reported to be involved in experimental sexting and 18% to have been involved in aggravated sexting. Thus, the percentage of sexting behaviors among adolescents appears to be lower than that among young adults as sexting appears to increase with age Finally, regarding risky sexting, only a previous study conducted in Italy [13] investigated sexting under substance use and sexting with strangers met only online among adolescents and young adults. Findings showed that 33% of participants involved in sexting during substance use at least once and the 1.9% shared sexts with strangers met only online at least once. Another research conducted in Sweden showed that 8% of adolescents between 12 and 16 years old shared sexts with a stranger [49]. Another study conducted in UK found that the 33% of sexual minorities young people shared sexts to a stranger [50]. However, there are no review of the literature or meta-analyses that compared percentages of risky sexting in different countries.

### 1.1. Personality Traits and Sexting

The specific relationship between personality traits and sexting among adolescents and young adults has been scarcely investigated. There is some evidence that personality traits are related to sexting behaviors. Different personality theoretical frameworks have been tested, including the Five Factor Model (e.g., [10,42,51]) and the HEXACO model of personality [15]. With regard to Big Five personality traits (that is, Openness, Conscientiousness, Extraversion, Agreeableness, Neuroticism) and general sexting behaviors among adolescents and young adults, results from different Western countries (i.e., America, Nigeria, and Spain) found that sexting was related to certain personality traits. In an American sample, extraversion and neuroticism were found to be positively related to sexting, whereas agreeableness was not [52]. Meanwhile, sending and receiving sexts were both related to higher scores of extraversion in a Nigerian sample [53]. Sexters reported higher levels in extraversion and neuroticism, and lower levels of agreeableness and conscientiousness compared to non-sexters in a Spanish sample [54]. From a longitudinal perspective, higher levels of extraversion and lower levels of agreeableness and conscientiousness increased the engagement in sexting a year later in a Spanish sample [12,42].

With respect to the HEXACO model of personality, which includes six broad dimensions of personality (that is, Honesty–Humility, Emotionality, eXtraversion, Agreeableness, Conscientiousness, and Openness to experience), only one study has investigated the relationship between personality traits and sexting as multidimensional construct, distinguishing experimental, aggravated, and at-risk sexting behaviors [15]. In a large sample of adolescents and young adults across ten different countries, Morelli and colleagues [15] found that higher scores in honesty-humility and conscientiousness negatively predicted all the different sexting dimensions. Emotionality and extraversion were positively related to sending own sexts, while agreeableness was negatively related to risky sexting. Finally, openness to experience was negatively connected to sharing sexts without someone else’s consent and sexting under pressure.

These results highlighted that personality traits may be predictors of different kinds of sexting. However, previous research has considered mainly adaptive personality traits as antecedents of sexting behaviors using broad and widely used personality taxonomies (i.e., Big Five and HEXACO), while maladaptive personality traits have been relatively overlooked [55].

### 1.2. The Dark Triad Traits

Following an evolutionary perspective on the development of personality traits, Buss [56] noted that traits emerged within the social context to which human beings had to adapt and focus on traits that have permitted people to satisfy evolutionary needs (for instance, security within the group). While some individuals faced evolutionary steps through prosocial means, therefore striving to be agreeable, conscientious, and honest, still others relied on more individually oriented approaches, including socially aversive strategies [57]. These latter strategies were connected to maladaptive personality traits.

Within the theoretical domain of maladaptive personality traits, the dark triad model has lately emerged as one of the most used taxonomies, measuring three specific traits: Machiavellianism, psychopathy, and narcissism. Together, these three traits represent the “Dark Triad” (from now on DT) of personality [58]. These traits share important aspects such as their social undesirable nature, similar phenotypical behaviors (e.g., manipulation), and conceptual similarities (e.g., egocentricity) [59]. DT traits describe individuals that share tendencies to be callous, selfish, and malevolent in their relations [60]. However, although intercorrelated, literature showed that each trait of DT represents a separate domain (e.g., [61,62]) showing differences, within a nomological networks, regarding their biological bases, underlying processes and dynamics, and association patterns with other constructs [63].

More specifically, psychopathy is marked by: (a) A lack of empathy for other people; (b) relations that are emotionally shallow; (c) little concern for social regulatory mechanisms; (d) impulsivity; and (e) a lack of guilt or remorse when actions are taken to harm others [64]. This dark trait refers to deviant behaviors with a short-term action for immediate gratification [61]. Psychopaths have very low regard for others, extreme irresponsibility of their actions toward people, and low levels of empathy (e.g., [65]). They are callous, emotionally cold, unable and unwilling to experience infatuation with another. Reactions to psychopaths’ individuals may be two-fold. At first, they may arouse interest from others, until their antisocial behaviors reveal themselves and they are eventually judged negatively. Conversely, sometimes they immediately create a repulsion caused by their unpleasant and impulsive behaviors. This dark personality trait has been empirically linked to negative outcomes such as various forms of criminality, including sexual assault [66], and it also predicts future sexual aggression among adults (e.g., [67]).

Narcissism is characterized by an inflated view of self; fantasies of control, success, and admiration with a driving motive behind callous behavior of self-love reinforcement [68,69,70]. Narcissists have been found to engage in denigration others [71], while aggrandizing the self as possible route to ego-reinforcement. A person with high levels of narcissism tends to exaggerate his or her achievements, he/she is hypersensitive to criticism, refuses compromise, and seeks out interpersonal and romantic relationships only with admiring individuals [72]. Narcissists appear generally popular, charming, and liked at first glance. However, this positive view might decline as interactions and relationships become deeper (e.g., [73]), because narcissists often tend to become arrogant, self-promoting, aggressive, and in general less amiable [74]. The negative outcomes related to narcissism are empirically related both to aggressive behaviors that usually occur when ego is threated, and to troubled romantic relationships due to egocentrism and infidelity [75].

Finally, Machiavellianism is defined by three sets of interrelated personal values: belief in the use of manipulative tactics in dealing with other people, a cynical view of human nature, and a moral conduct that puts personal convenience above all principle. Machiavellians view others in an adverse way: in their cynical worldview, people are weak, fallible, and manipulable [61,63,76]. Research found that younger Machiavellians appear to be liked (e.g., [77]); however, people judge Machiavellians more negatively after prolonged interactions [78]. These negative evaluations may stem from them taking revenge against others [79] and lying more regularly to their friends [80].

As highlighted by the literature reviewed above, the DT traits are generally associated with a disposition to engage in antisocial behaviors to attain one’s own goals [81]. Particularly, taken together, these traits have been associated with more high risk sexual behaviors, including coercion [82], more positive attitudes toward rape [83], repeated sexual advances [84], a greater propensity to commit romantic revenge [85], and greater enjoyment of tormenting others online [86]. DT has also been found to be related to both perpetration and victimization of bullying [87].

One study showed that DT traits are related to sexting behaviors and found a significant relationship between all three DT traits and sext dissemination [88], with Machiavellianism being the strongest predictor. Other authors found that Machiavellianism was a positive predictor of unsolicited explicit images [89], narcissism predicted higher levels of social activity in the online community and more self-promoting content in several aspects of the social networking [74], while psychopathy was associated with risky behavior, including antisocial sexual activities [90]. As a matter of fact, literature investigating the relationship between the DT traits and sexting, in its multifaced expressions, appears limited and in need of further exploration.

### 1.3. Aim and Hypotheses of the Present Study

The main goal of the present study was to examine the extent to which the DT personality traits (i.e., Machiavellianism, narcissisms and psychopathy) are related to diverse kinds of sexting behaviors: experimental (i.e., sending own sexts), risky sexting (sexting during substance and alcohol use and sharing sexts with strangers that were met online), aggravate sexting (for perpetration: sharing sexts of someone else without his/her permission; for victimization: sexting under pressure), among adolescents and young adults across different cultures. Recently, a growing literature has begun to show that dark personality factors are linked to different online behaviors [91]. Nevertheless, to our knowledge, there are no studies yet that have explored the relationship between the DT personality traits as antecedents of different kinds of sexting across countries. In this sense, the present paper would represent the first empirical attempt to fill this gap in our knowledge.

There are two main reasons why we expect these three personality traits to affect different sexting behaviors. Previous research has shown that a variety of personality traits are important factors affecting online behaviors (e.g., [92]). For instance, aspects related to maladaptive personality traits of narcissism and psychopathy, such as exhibitionism and lack of empathy, frequently emerge in the online context [93,94]. Second, personality characteristics affect the way individuals behave and deal with online relationships. In this perspective, certain maladaptive personality dispositions may lead individuals to behave more negatively, affecting online sexual communication and more likely leading to problematic sexting behaviors and subsequent negative consequences.

A particularly innovative element of the present paper is the investigation of possible antecedents related to different kinds of sexting in different countries that permits us to gain a deeper understanding and generalization of the phenomenon. There are only two cross-cultural studies that have investigated the relationship between personality traits as antecedents of sexting. The first one, involved ten western and non-western countries and considered the HEXACO personality traits as predictors of different kinds of sexting behaviors, in adolescents and young adults [15]. The second cross-cultural study was conducted in 20 European countries and focused on sensation seeking as personality predictor of the posting of sexual messages of any kind on the Internet [46]. However, no study has previously investigated the relationships between DT traits and sexting across different countries at the same time.

Moreover, there are substantial differences between the aforementioned studies and the current study. First, both papers focused on the role of adaptive personality traits as predictors of sexting [15,46]. The role of maladaptive personality traits was not investigated. Furthermore, only the study of Morelli and colleagues [15] examined different types of sexting at the same time, namely own sexts, non-consensual, and risky sexting. On the contrary, the study of Baumgartner and colleagues [46] assessed sexting as a unidimensional construct, using a single item measure. A limitation in measuring a construct with a single item relates to the goodness of psychometric assessment in terms of validity and reliability [95]. In this perspective, it is assumed that a multi-item measure as the one employed in the present research would be able to better represent the complexity of different kinds of sexting. Following this line of reasoning, in every country involved in the present study, the same constructs definitions, the same multi-item measure for assessing sexting behaviors [15,40,96,97], and the DT personality traits [57], were applied. This methodological approach enabled us to overcome possible issues related to the cross-country comparison.

To sum up, the present study represents an effort to gain a deeper understanding of the effect of DT personality traits on different kinds of sexting. More specifically, the current paper aims to contribute to this growing area of research in five ways. First, this study enhances the research on individual-level antecedents of sexting behaviors by investigating its relationship with maladaptive traits of personality as Machiavellianism, narcissism, and psychopathy, which will contribute to the understanding of the phenomena in a relatively understudied domain. Second, the present study is the first that investigates different kinds of sexting in relation to DT personality traits from 11 countries, across four continents. Third, the current study uses a solid measure to evaluate the frequency during the past year of different kinds of sexting via a reliable and valid instrument, named as Sexting Behaviors Questionnaire [13]. Fourth, related to previous point, this study investigates a plurality of sexting behaviors, distinguishing between experimental, aggravated, and risky behaviors. Finally, this study is based on a well-established taxonomy for examining maladaptive personality traits named the Dark Triad, that is Machiavellianism, narcissism, and psychopathy.

Regarding the relationship between DT personality traits and different sexting behaviors, in line with results from previous studies [88,89], it was expected that higher levels of Machiavellianism, psychopathy, and narcissism would be associated with higher likelihood of engaging in experimental sexting (i.e., consensual exchange of own sexts), aggravated sexting (i.e., for perpetration: non-consensual sexting; for victimization: sexting under pressure), and risky sexting (i.e., sexting during substance and alcohol use and sharing sexts with strangers that were met online).

## 2. Materials and Methods

### 2.1. Participants and Procedure

Data used in the present study were part of a larger cross-countries project on sexting. Data collection involved 11 countries (namely, Belgium, China, Czech Republic, Ireland, Italy, Malaysia, Poland, Russia, Turkey, Uganda, and USA) resulting in a total of 6093 participants (3682 girls and 2401 boys), with average age of 20.35 (SD = 3.63) ranging from 13 to 30 years old. Regarding relationship status, about 81.8% (*n* = 4983) reported currently having a dating partner or having had one in the past, while the remaining 17.5% (*n* = 1069) reported that they had never had a dating partner. Descriptive statistics of participants for each country are reported in Table 1. The participants of the samples of each country were independent to each other and no one was measured repetitively.

To assess the sample size required for each country, in order to attain enough statistical power and reduce the occurrence of Type II Error, an a priori power analysis was conducted [98]. At the bivariate level we set the following parameters: a small to medium effect size was hypothesized (r = 0.20), alpha level was fixed to 0.05 and power to 0.80 [98]. The result of the power analysis pointed out a required sample size of minimum 194 participants for each country. Therefore, scholars of each country were asked to collect at least more than 200 participants. Therefore, the global sample size of 6093 is to be considered more than adequate in terms of statistical power.

Researchers of each country were contacted by the Italian group that coordinated the entire project. If they accepted to join the project they had to sign a scientific agreement in which all information about requested sample size, characteristic of the sample, and procedure were reported. An English version of the questionnaire was shared with the researchers from all involved countries and, excepting for English spoken countries, each group worked to a language adaptation of the survey, using a procedure of translation and back translation. Data were collected by all countries between the year 2017 and the year 2018. The study was conducted according to the guidelines of the Declaration of Helsinki, and approved by Ethics Committee of the Department of Developmental and Social Psychology, Sapienza University of Rome (protocol code 405, 11/23 and 07.22.2015).

A survey online was completed by all participants. Underage participants were recruited in public schools and, after having obtained written informed consents from their parents, they completed the online survey in the informatic lab of the schools. Participants over 18 year old were collected in university and through a snowball sampling. University students were asked to share the link of the survey among their social networks’ contacts. Each participants gave his/her own consent at the beginning of the survey online, by clicking on the button “yes, I accept to participate to this study.” The participants were told that the survey was totally anonymous and the online compilation of the questionnaires guaranteed greater respect for privacy, given the very sensitive and intimate nature of the data requested. Among all the participants reached, only the questionnaires fully filled were considered valid. Thus, the response rates for each countries are as follows: 93% for Belgium, 90% for China, 85% for Czech Republic, 91.5% for Ireland, 91% for Italy, 91% for Malaysia, 100% for Poland, 100% for Russia, 99% for Turkey, 85% for Uganda, and 98% for USA.

### 2.2. Measure

Participants reported their age, biological sex (girls were coded as 0; boys as 1), and dating relationship status (having never had a partner was coded as 0; currently have or have had a partner was coded as 1).

Sexting was conceived as sending or receiving sexually suggestive or provocative messages/photos/videos via mobile phone and/or Facebook or other Internet social networking site. The scale measured the frequency of experimental sexting (i.e., consensual exchange of own sexts), aggravated sexting (operationalized in two different dimensions: non-consensual sexting for perpetration and in sexting under pressure for victimization), and risky sexting (i.e., sexting during substance and alcohol use and sharing sexts with strangers that were met online). Thus, the frequency of these different kinds of sexting behaviors, in which participants engaged during the last year, was assessed by 18 items taken from the Sexting Behaviors Questionnaire (SBQ) [13]. Participants rated each item of the questionnaire on a 5-point Likert scale from 1 = Never to 5 = Always or almost daily. The dimension of sharing own sexts was assessed by four items asking how often participants had privately sent and publicly posted their own sexts (Cronbach’s alpha of 0.72). Eight items measured the dimension of non-consensual sexting, consisting of privately sending and publicly posting sexts of someone else (i.e., a partner or an acquaintance) without his/her consent (Cronbach’s alpha was 0.93). Four items tapped the dimension of risky sexting, which comprised engaging in sexting during substance and alcohol use and sharing sexts with strangers that were met online (Cronbach’s alpha of 0.72). Finally, two items assessed sexting under pressure of a partner or friends (Cronbach’s alpha was 0.69). Reliabilities of sexting dimensions for each country are reported in Table 2. We checked that factor structure of the SBQ was replicated across countries, running a multi-group model. The configural invariant model across countries exhibited a acceptable fit, chi-square (20) = 697.58, *p* < 0.001, CFI = 0.92, SRMR = 0.05, suggesting that the overall factor structure holds up reasonably similar for all countries. The items of each dimensions of the SBQ are reported in Appendix A Table A1.

The Dark triad traits were measured by mean of the 12-item Dark Triad Dirty Dozen scale [57,99]. This scale evaluated the three socially undesirable dimensions of personality on a 9-point Likert scale from 1 (Strongly disagree) to 9 (Strongly agree), with four items for each dimension: Machiavellianism (a sample item is “I tend to manipulate others to get my way”), narcissism (a sample item is “I tend to want others to admire me”), and psychopathy (a sample item is “I tend to be callous or insensitive”). The three dimensions showed a good reliability (Cronbach’s alpha of 0.85 for Machiavellianism, 0.87 for narcissism, and 0.78 for psychopathy). Reliabilities of dark triad traits for each country are reported in Table 3. We tested whether the factor structure of the Dark Triad Dirty Dozen scale was replicated across countries. The findings of the configural invariant model across countries highlighted a good fit, chi-square (330) = 1279.79, *p* < 0.001, CFI = 0.97, SRMR = 0.02, indicating that the overall factor structure holds up similarly for all countries.

### 2.3. Data Analysis

First, descriptive statistics, frequencies and correlations among variables were computed. Afterwards, we investigated how the Dark Triad traits (i.e., Machiavellianism, narcissism, and psychopathy) predicted different sexting behaviors (i.e., sending own sexts, risky sexting, non-consensual sexting, sexting under pressure), controlling for biological sex and age. As participants were nested in different countries, we run a linear mixed model for each of the four dependent variables (i.e., sexting) in which Country was the grouping variable. In our model, as fixed effects predictors, we had the two demographical variable, age (in years), biological Sex (0 = female, 1 = male), and the three dark traits (i.e., Machiavellianism, psychopathy, and narcissism), plus a fixed intercept, and one random intercept for each of the country. Furthermore, we also considered the possible interactions between the demographical variables and the DT traits, so we added to the model the interaction terms as six more fixed effects, namely age*Machiavellianism, age*psychopathy, age*narcissism, Sex*Machiavellianism, sex*psychopathy, sex*narcissism. Following suggestions of Aiken and West [100] variables were mean centered. To interpret the findings of possible interactions between variables, a simple slope analysis was conducted.

Since non-consensual sexting and sexting under pressure contained items about sexting behaviors with a dating partner, the analyses for these variables were run only on the subsample of participants who reported to have or have had a dating partner. Moreover, data from Malaysia regarding risky sexting and sexting under pressure were not available. Therefore, in these cases the analyses were run without Malaysia. The exact number of observations for each of the analysis will be indicated in Tables 4, 5, 7, 9, and 11. Data are available for inspection under request: The first author will provide clarification if needed.

## 3. Results

### 3.1. Descriptive and Correlations

First, the prevalence of each sexting behavior is reported. The 39.7% (*n* = 2418) reported to have sent own sexts at least once, the 43.2% (*n* = 2503) reported to have engaged in risky sexting at least once, the 16.6% (*n* = 829) reported to have sent a sext of somebody else without consensus at least once, and the 15.7% (*n* = 750) reported to have sent sexts at least once under pressure of a partner or friends. Detailed descriptive statistics by countries are displayed in Table 2. In Table 3 are reported detailed statistics by country also for the three dark triads dimensions.

Correlations, means, and standard deviations of investigated variables are reported in Table 4. The three dark traits were significantly and positively correlated to all studied sexting behaviors.

### 3.2. Dark Traits and Sexting

As stated before, four linear mixed models were run in order to investigate how Dark personality traits (i.e., Machiavellianism, Psychopathy, and Narcissism) predicted different sexting behaviors, namely sharing own sexts, risky sexting, non-consensual sexting, and sexting under pressure, controlling for biological sex, and age. Moreover, we added to the model the interaction terms between the demographical variables and the DT traits.

The first mixed model regarded sharing own sexts, which accounted for the 5.2% of the variance. Results of the analysis were reported in Table 5 and Table 6. Biological sex did not have a significant effect, while age was a significant predictor with older participants that tended to share more their own sexts. Machiavellianism and narcissism emerged as significant predictors (Table 5): participants who scored higher on these two traits were more likely sharing their own sexts. Machiavellianism emerged as the best predictor.

**Figure 1:**
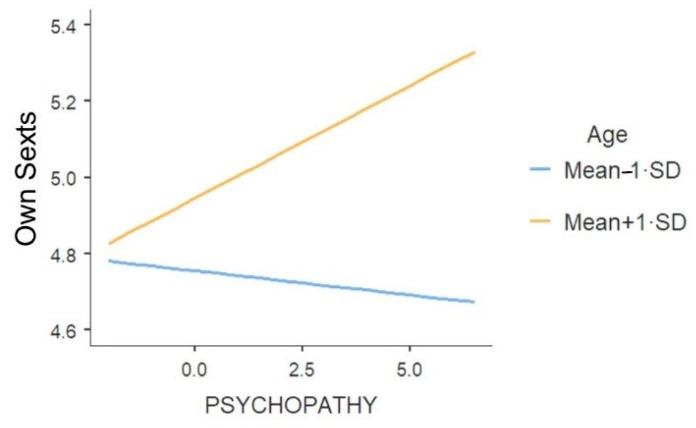
The effect of psychopathy on sharing own sexts in function of age.

Interestingly, a significant interaction occurred between age and psychopathy (Table 5). To interpret this interaction effect, a simple slope analysis was performed. When the level of age was low (Mean-1·SD), the effect of psychopathy on sharing own sext was not significant, *B* = −0.01, *se* = 0.02, *t* = −0.55, *p* = 0.58. On the contrary, when the level of age was low (Mean + 1·SD), the effect of psychopathy on sharing own sext turned out to be significant, *B* = 0.06, *se* = 0.02, *t* = 2.58, *p* = 0.01. It appeared that higher scores of psychopathy were related to more sharing of own sext only for older participants (see Figure 1).

The second model considered risky sexting, which explained 11% of the variance. Results of the analysis were reported in Table 7 and Table 8. Both biological sex and age were significant predictors, with boys and older participants that exhibited a tendency to do more risky sexting (Table 7). Machiavellianism and psychopathy emerged as significant predictors (Table 7): participants who scored higher on these two traits were more likely to do risky sexting. Again, Machiavellianism came out as the best predictor. No significant interaction was found between the socio-demographical variables and the DT traits.

The third model concerned non-consensual sexting, which explained 11% of the variance. Results of the analysis were reported in Table 9 and Table 10. Both biological sex and age were significant predictors, with boys and younger participants doing more non-consensual sexting (Table 9). Both Machiavellianism and psychopathy were significant predictors (Table 9): participants who scored higher on these two traits reported more non-consensual sexting behaviors. In this case, psychopathy emerged as the strongest predictor of non-consensual sexting.

**Figure 2:**
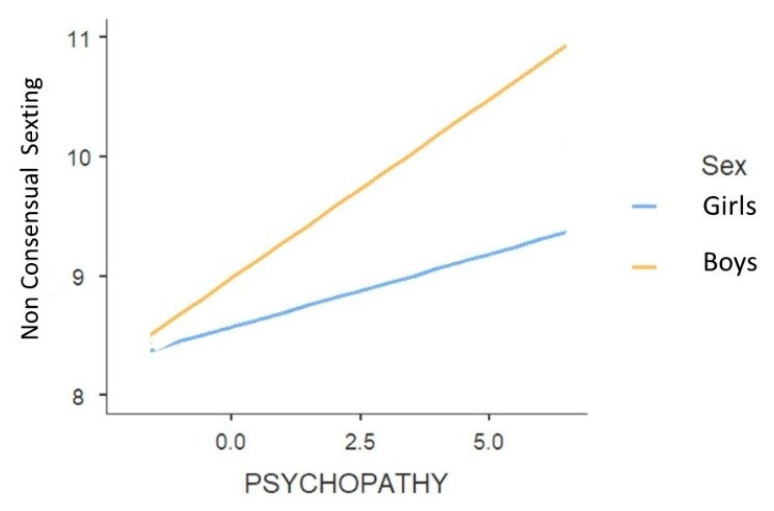
The effect of psychopathy on non-consensual sexting in function of biological sex.

Two significant interactions were found between biological sex and psychopathy and between age and narcissism (Table 9). To interpret these interaction effects, two simple slope analyses were run. As regard biological sex and psychopathy, the relationship between psychopathy and non-consensual sexting was stronger for boys, *B* = 0.30, *se* = 0.04, *t* = 8.16, *p* < 0.001, than for girls, *B* = 0.12, *se* = 0.04, *t* = 2.91, *p* = 0.004 (see Figure 2).

**Figure 3:**
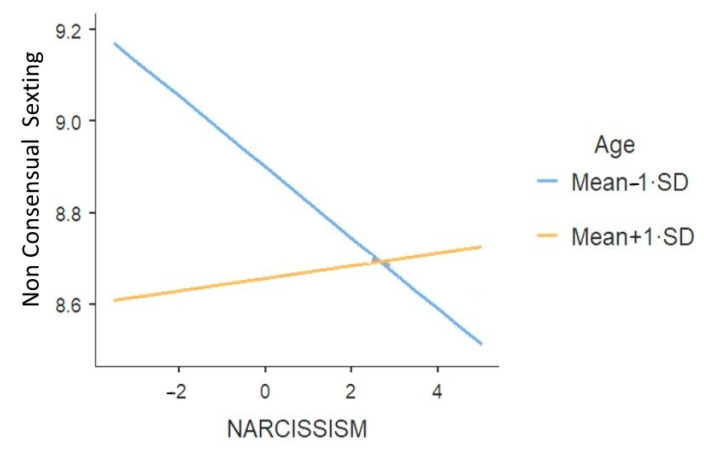
The effect of narcissism on non-consensual sexting in function of age.

A significant interaction was also found between age and narcissism (Table 9). The relationship between narcissism and non-consensual sexting was significant and negative for younger participants (Mean-1·SD), *B* = −0.08, *se* = 0.03, *t* = −2.57, *p* = 0.01, while it was non-significant for older participants, *B* = 0.01, *se* = 0.03, *t* = 0.40, *p* = 0.65. In this case, it appeared that higher scores of narcissism were negatively related to non-consensual sexting for younger participants (see Figure 3). To put it in other terms, younger narcissistic participants tended to do less non-consensual sexting.

The last fourth and last model focused on sexting under pressure, accounting for 10.5% of the variance (see Table 11 and Table 12 for results). The demographical variables, biological sex and age, were significant predictors, with boys and younger participants engaging more in sexting under pressure (Table 11). Again, Machiavellianism and psychopathy were significant predictors (Table 11): participants who scored higher on these two traits reported of doing more sexting under pressure. In this case, Machiavellianism and psychopathy emerged as predictors of sexting under pressure with equal strength.

**Figure 4:**
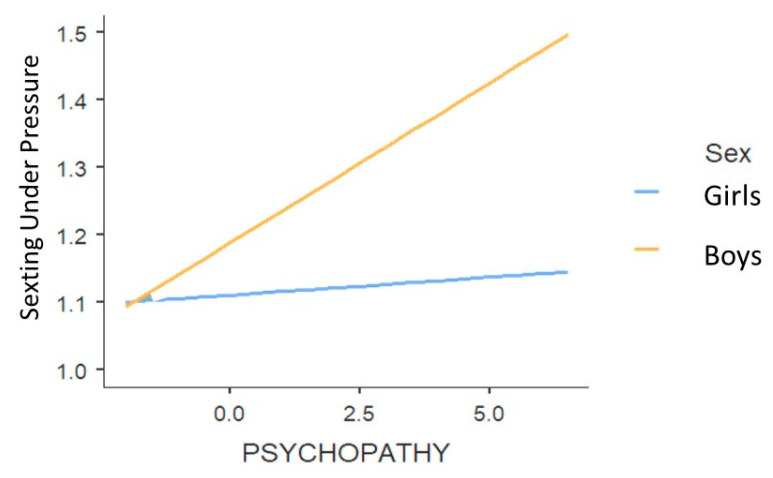
The effect of psychopathy on sexting under pressure in function of biological sex.

Two significant interactions were found between biological sex and psychopathy and between age and Machiavellianism (Table 11). To interpret these interaction effects, two simple slope analyses were run. As regard biological sex and psychopathy, the relationship between psychopathy and sexting under pressure was stronger for boys, *B* = 0.05, *se* = 0.006, *t* = 7.32, *p* < 0.001. For girls, this relationship was non-significant, *B* = 0.005, *se* = 0.007, *t* = 0.71, *p* = 0.48. In this regard, it seemed that boys with higher scores on psychopathy were doing more sexting under pressure, while in girls there was no relation between psychopathy scores and doing sexting under pressure (see Figure 4).

**Figure 5:**
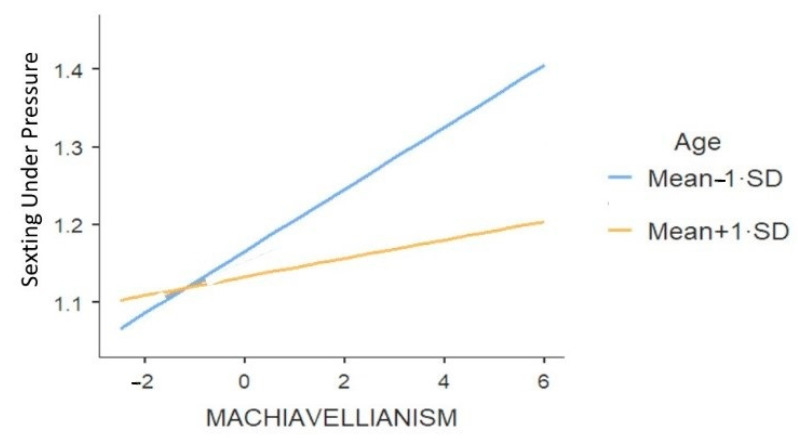
The effect of Machiavellianism on sexting under Pressure in Function of Age.

Another significant interaction emerged between age and Machiavellianism (Table 11). The relationship between Machiavellianism and sexting under pressure was significant and positive for younger participants (Mean-1·SD), *B* = −0.08, *se* = 0.03, *t* = −2.57, *p* = 0.01, while it was non-significant for older participants, *B* = 0.01, *se* = 0.03, *t* = 0.40, *p* = 0.65. It appeared that higher scores on Machiavellianism were related to sexting under pressure in younger participants but not in older ones (see Figure 5). Framed differently, younger Machiavellians tended to engage in more sexting under pressure than older Machiavellians.

## 4. Discussion

The present study investigated how specific maladaptive personality traits related to different kinds of sexting among adolescents and young adults, across 11 different countries that cover four continents (Europe, Asia, Africa and America). The main aim was to identify specific relationships between the Dark Triad (DT) traits, and different kinds of sexting behaviors: experimental sexting (i.e., consensual exchange of own sexts), aggravated sexting (i.e., for perpetration: non-consensual sexting; for victimization: sexting under pressure), and risky sexting (i.e., sexting during substance and alcohol use and sharing sexts with strangers). All the investigated relationships were analyzed considering the possible effect of biological sex and age.

Regarding biological sex, results showed that boys were more likely to be involved in risky sexting and in both forms of aggravated sexting (i.e., non-consensual sexting and sexting under pressure). These findings partially confirmed results on sex differences in sexting underlined by a recent meta-analyses that found males more involved in non-consensual sexting than females [2]. Conversely with respect to a recent study [16], our results found that males seem to be involved also in more sexting under pressure. Moreover, previous literature suggested that males are more likely to be involved, in general, in risky behaviors than females probably due to high levels of sensation seeking and impulsivity [101], explaining why boys could report also more risky sexting.

Regarding age differences, results showed that older participants are more likely to involve in sharing own sexts and risky sexting than younger ones. These results are in line with previous studies [1,22] that suggested how sexting increase with age similarly to the developmental tendency of sexual activity [102]. On the contrary younger participants reported more both forms of aggravated sexting (i.e., non-consensual sexting and sexting under pressure) than older ones. These results could be related to the fact that adolescents are less oriented toward the future and show less considerations for future consequences that lead them to involve in more aggravated forms of sexting [103,104].

The DT traits have been considered as socially undesirable by various authors due to superiority and dominance (i.e., narcissism), glib social charm and manipulativeness (i.e., Machiavellianism), and callous social attitudes and impulsivity (i.e., psychopathy) [58,105]. To date, few studies have provided empirical evidence that the DT traits exhibit relationships with specific types of sexting behavior. In particular, Clancy and colleagues [55] showed that individuals who have disseminated sexts had higher scores in Machiavellianism, narcissism, and psychopathy. March and Wagstaff [88] found that Machiavellianism, narcissism, and psychopathy significantly correlated with the enjoyment of sending explicit pictures of own genitals to other people. However, no study has yet considered the relationships of DT traits with different forms of sexting behaviors simultaneously and, to the best of our knowledge, there is no other research that investigated these variables in the same model across different countries. In sum, the present study provided empirical evidence that different kinds of sexting behaviors, that is sent and publicly posted own sexts, risky sexting (i.e., engage in sexting during recreational drugs and alcohol use, share sexts with strangers met online), non-consensual sexting (i.e., share sexts from someone else without his/her consent), and sexting under pressure (i.e., being forced by someone, such as partner or friends, to share sexts) were predicted by different DT personality traits (i.e., Machiavellianism, narcissism and psychopathy).

Narcissism and Machiavellianism emerged to be positive predictors of sharing own sexts. These results were in line with the previous studies that investigated online behaviors of narcissists (e.g., [93]) and Machiavellians [89]. In fact, individuals with high narcissism generally display more online behaviors geared toward self-presentations, tend to use pictures and words to communicate about themselves more frequently and, mostly, in positive ways [74]. With regard to the relationship between sharing own sexts and Machiavellianism, our results somewhat aligned with previous studies [88,89]. Moreover, research showed that individuals with high Machiavellianism are behavioral strategists, effectively and charmingly exploiting situations and people for their own benefit [106,107]. Prior qualitative work found that some sexters would send a sexting image of themselves first in order to pressure the receiver to respond with an image [108]. Future research could investigate whether certain personality traits could make individuals more likely to use sexting in this way. It is worthy to note that psychopathy showed no main effect on sharing own sexts. However, it appeared that this relationship was conditioned by age: specifically, the relationship between psychopathy and sharing own sexts was stronger for older participants.

Both risky sexting (i.e., sexting during substance and alcohol use and sharing sexts with strangers), and aggravated sexting (i.e., non-consensual sexting and sexting under pressure), were positively predicted by both Machiavellianism and psychopathy. These results will be described, separately, below.

Machiavellianism emerged as a positive predictor of risky sexting. The possible explanation of this empirical evidence can be found in some aspects of Machiavellian trait, which is characterized by manipulation and deception of others, combined with a lack of emotionality and disregard of morality [105]. Indeed, previous studies showed evidence of the strategic nature of Machiavellianism [61] and increased manipulative behavior and sexual coercion [109]. Our findings showed that risky sexting was positively predicted by psychopathy as well. These findings were in line with previous research that showed a strong association between psychopathy and perpetration of sexually deviant behaviors, including forms of online sexual harassment [110]. People with high levels of psychopathy are more inclined to unrestricted attitudes toward promiscuity, and especially a lack of attachment [57]. Thus, it may be appropriate for them to share sexts with strangers they met online. Moreover, people with higher psychopathy levels are very impulsive, showing low levels of fear and self-control (e.g., [111]), and it is very likely that they could engage in sexting during substance and alcohol use.

Non-consensual sexting was positively predicted by Machiavellianism. Machiavellian people have emotional detachment and low empathy with a willingness to exploit others [76,78,112]. Moreover, they adopt strategies with long-term orientations in making decisions and planning behaviors to reach their personal purposes [61]. Thus, they may act planning a strategy by getting and posting sexts of someone else (a partner or an acquaintance) without his or her consent.

Psychopathy emerged as positive predictor of non-consensual sexting. Earlier studies have shown that sending sexts can also be a part of a risky sexual behavior (e.g., [113,114]). In this regard, people with high psychopathy have little empathy for other people and no concern for behaviors that are not considered socially acceptable [115]. Therefore, a lack of care, callousness, and lack of empathy could be the reasons why people high in psychopathy, with socially aversive behaviors, participate in non-consensual sexting by privately sending and publicly posting sexts of someone else without consent. This kind of sexting behavior, in fact, shows very low regard for others and extreme irresponsibility of actions toward other people involved in sexting. Individuals high in psychopathy can also make non-consensual sexting as they do not care to do harm or cause distress, and this trait often represents the best predictor of different form of romantic revenge [86,116,117].

Sexting under pressure was positively predicted by Machiavellianism and psychopathy. Previous studies explored sexting under pressure, and found that it may be due to influence of the peer group (e.g., [118]) or of partners, with girls (vs. boys) reporting more perceived pressure to sext from partners (e.g., [119]). Individuals high in Machiavellianism are mostly concerned about maintaining a positive image within the group [61]. Thus, it is possible that they are involved in sexting under pressure thinking that this behavior would maintain their popularity within the group. In regards to psychopathy, research found that, conversely to Machiavellians, people high in psychopathy tend to act instinctively and without worrying about their reputation.

The relationship between psychopathy and both forms of aggravated sexting (i.e., non-consensual sexting and sexting under pressure) was stronger for boys than girls. Moreover, it is worthy to note that narcissism showed no main effect on non-consensual sexting. However, it appeared that this relationship was conditioned by age: specifically, the relationship between narcissism and non-consensual sexting was negative for younger participants. Finally, the relationship between Machiavellianism and sexting under pressure was stronger for younger participants.

Overall, our findings suggest that not all DT traits are equally related to all kinds of sexting behaviors. Indeed, the present study is the first attempt to provide empirical evidence that different maladaptive personality traits predicts different kinds of sexting behaviors. In fact, sharing own sexts was predicted by narcissism and Machiavellianism, whereas risky sexting, non-consensual sexting and sexting under pressure were related to psychopathy and Machiavellianism. Narcissism did not appear to be related to more negative form of sexting, which were predicted by psychopathy instead. Ultimately, Machiavellianism represented a significant predictor of all form of sexting behaviors.

In sum, our findings add to the research on sexting, as they help deepen the knowledge how individual differences through maladaptive personality traits, are involved in sexting behaviors.

### 4.1. Limitation and Future Directions

The present study does have limitations. First, non-probabilistic sampling limited somehow the generalizability of our results. Participants within each country were selected mainly via a snowballing procedure, and therefore the sample cannot be considered as representative of adolescents and young adults of all countries included in the study. Further research on larger samples within each country would be necessary to test the generalizability of our results.

Second, the cross-sectional nature of data does not allow us to draw casual inferences among variables. Although measurement at different time points represents a necessary but not sufficient condition for assessing causality [120], and the use of longitudinal design as a remedy to solve the issue of causality is often overstated [121], future longitudinal studies to replicate our results are needed.

Third, self-reported nature of the measure used in this study may represent an evident limit. Being sensitive and private topics, they may be biased due to social desirability in the answers which, considering the nature of the topic, could be expected. Moreover, especially younger participants may have difficulties in correctly estimating their behaviors. Additionally, respondents in some countries with a less liberal or more traditional culture could under-report information on sexuality.

Last, the DT personality traits accounted a certain amount of variance (from 4.7% to 10.5%), for different sexting behaviors as dependent variables. Therefore, results regarding the relationship between the DT personality traits and different kinds of sexting considered, should be interpreted with caution as it is possible that other variables might explain these kinds of behaviors. This could be a valuable starting point for future studies.

### 4.2. Implications

Despite these limitations, some practical and theoretical implications can be drawn from the present study. Starting from the latter, prior research showed a significant gap in the current literature around maladaptive personality traits and sexting behaviors, particularly with regard to an absence of studies on this relationship conducted in different countries at the same time. This study was aimed to address this gap involving adolescents and young adults from 11 countries in four continents, and their rates of different kinds of sexting, and the association with the DT personality traits. We found significant differences in the association with each DT personality traits, which has several implication. From a theoretical perspective, our results suggest that different kinds of sexting behaviors are predicted by specific maladaptive personality traits that should be taken into account in future research, with regard to determining the impact of engagement in specific kind of sexting behaviors. Our findings shed a new light on the differences and equalities from western to non-western populations of young people on the variables investigated. Related to previous point, using the same multi-item questionnaire across all countries involved in the present study, represents a strength of the study, instead of using a single-item measure used in previous cross-countries studies [122,123]

From a practical point of view, findings of the present cross-country study are relevant for education and prevention programs, as they demonstrate the importance of identifying certain sexting behaviors as a vehicle to abuse or harm others. These programs should aim in teaching young people to avoid non-consensual sexting, and also deter individuals from acting aggressively and exploiting a sexual/romantic relationship for secondary purposes. Educators and psychologists should provide programs for young people focused on both learning general healthy relationship skills, which will help them to positively interact with others also in an online context, and on digital citizenship, including information of potential negative consequences of sexting, increasing their awareness of this phenomenon. Additionally, they should support programs to help young people to manage negative consequences caused by being a perpetrator or a victim of aggravated sexting.

Finally, these results have some implications for professionals working in psychotherapy centers or school setting. From a clinical point of view, early screenings of maladaptive personality traits on young people could provide a profile to identify who tends to engage in risky and aggravated sexting behaviors. Informing these high-risk individuals of the possibility of negative consequences of specific kinds of sexting may be a tool for decreasing engagement in harmful sexting behaviors for adolescents and young adults.

## 5. Conclusions

This study shed a new light on adolescents and young adults sexting behaviors across 11 countries of four continents. Furthermore, it investigated simultaneously for the first time the relationship between the Dark Triad (DT) traits, and different kinds of sexting behaviors across 11 different countries. The results are supported by a validated measure of sexting, providing solid theoretical explanations on the relationship among variables considered in this study. Our findings have noteworthy features both for theoretical and practical implications as they consider specific maladaptive personality traits as predictors of different kinds of sexting.

## Figures and Tables

**Table 1:** Sample characteristics by country.

		Age		Biological Sex		Dating Relationship	
Countries	Sample Size	Range	M(SD)	Girls	Boys	No	Yes
Belgium	505	14–30	19.17 (3.42)	344	161	93	412
China	361	17–30	21.27 (2.64)	220	141	106	252
Czech Republic	733	13–30	19.51 (3.16)	469	264	74	659
Ireland	271	13–17	15.05 (0.69)	0	271	113	158
Italy	805	13–30	20.85 (4.25)	474	330	82	722
Malaysia	305	14–30	22.09 (2.16)	229	76	88	217
Poland	1075	13–30	20.8 (4.18)	543	532	275	800
Russia	278	15–30	19.79 (3.31)	208	70	51	227
Turkey	601	18–30	22.65 (2.95)	419	176	65	535
Uganda	226	14–20	17.29 (1.31)	137	86	60	130
USA	933	18–30	20.74 (2.36)	639	294	62	871

Note. Few participants did not report their biological sex or dating relationship status.

**Table 2:** Descriptive statistics of sexting by country.

	Sexting Behaviors															
	Own Sexts				Risky Sexting				Non-Consensual Sexting ^a^				Sexting under Pressure ^a^			
Countries	M (SD)	Range Min-Max	Sexters ^c^	α	M (SD)	Range Min-Max	Sexters ^c^	α	M (SD)	Range Min-Max	Sexters ^c^	α	M (SD)	Range Min-Max	Sexters ^c^	α
Belgium	1.18 (0.30)	1–3	41%	0.69	1.24 (0.32)	1–3	53.1%	0.54	1.02 (0.09)	1–1.88	10%	0.58	1.07 (0.22)	1–2.5	18.2%	0.52
China	1.12 (0.45)	1–4.25	14.4%	0.91	1.09 (0.38)	1–4.25	12.2%	0.87	1.14 (0.49)	1–4	16.3%	0.96	1.15 (0.54)	1–5	11.5%	0.91
Czech Republic	1.26 (0.51)	1–5	45.7%	0.76	1.40 (0.71)	1–5	54.2%	0.81	1.11 (0.44)	1–5	21.9%	0.94	1.32 (0.73)	1–5	28.7%	0.76
Ireland	1.23 (0.63)	1–5	28.4%	0.88	1.40 (0.85)	1–5	35.8%	0.87	1.19 (0.63)	1–5	24.7%	0.95	1.21 (0.75)	1–5	15.8%	0.91
Italy	1.24 (0.37)	1–4	47.3%	0.60	1.23 (0.41)	1–4	41.3%	0.52	1.07 (0.22)	1–3.63	18.6%	0.81	1.08 (0.28)	1–3.50	10%	0.50
Malaysia	1.20 (0.55)	1–5	25.2%	0.86	==	==	==	—	1.16 (0.53)	1–5	20.3%	0.97	==	==	==	—
Poland	1.25 (0.48)	1–5	40.6%	0.72	1.31 (0.54)	1–5	48.3%	0.71	1.07 (0.35)	1–5	14%	0.95	1.07 (0.38)	1–5	5.5%	0.84
Russia	1.23 (0.35)	1–3.25	45.7%	0.50	1.18 (0.30)	1–2.50	40.3%	0.51	1.06 (0.17)	1–2.38	19.8%	0.65	1.12 (0.33)	1–3	15%	0.50
Turkey	1.15 (0.29)	1–3	30.4%	0.54	1.30 (0.50)	1–4.25	42.8%	0.73	1.03 (0.11)	1–2.38	9.7%	0.66	1.36 (0.65)	1–5	35.5%	0.60
Uganda	1.15 (0.59)	1–5	15.9%	0.91	1.14 (0.52)	1–5	15%	0.85	1.26 (0.66)	1–4.25	24.6%	0.93	1.19 (0.56)	1–4	13.8%	0.60
USA	1.29 (0.40)	1–5	54.3%	0.61	1.29 (0.46)	1–3.25	45.9%	0.62	1.08 (0.29)	1–5	16.6%	0.90	1.07 (0.27)	1–3	8.3%	0.50

Note. ^a^ Statistics for non-consensual sexting and sexting under pressure were computed on participants who reported to have or have had a dating partner. ^c^ Sexters = percentage of participants who sexted at least once. α = Cronbach’s alpha.

**Table 3:** Descriptive statistics of dark triad traits by country.

	Machiavellianism			Psychopathy			Narcissism		
Countries	M (SD)	Range Min-Max	α	M (SD)	Range Min-Max	α	M (SD)	Range Min-Max	α
Belgium	3.67(1.68)	1–9	0.80	2.46(1.46)	1–7.25	0.79	4.07(1.87)	1–9	0.87
China	2.46(1.38)	1–7.25	0.86	2.57(1.45)	1–9	0.80	4.32(1.91)	1–9	0.89
Czech Republic	4.48(2.05)	1–9	0.84	3.09(1.71)	1–9	0.73	4.61(2.11)	1–9	0.87
Ireland	4.36(2.07)	1–9	0.78	3.33(1.79)	1–9	0.76	5.46(1.97)	1.9	0.81
Italy	2.68(1.80)	1–9	0.84	2.76(1.69)	1–9	0.72	3.90(2.17)	1–9	0.86
Malaysia	3.66(1.99)	1–9	0.90	3.50(1.79)	1–9	0.84	4.24(2.14)	1–9	0.90
Poland	3.78(2.04)	1–9	0.88	3.21(1.87)	1–9	0.81	4–76(2.21)	1–9	0.88
Russia	4.76(2.09)	1–9	0.88	3.36(1.77)	1–8.5	0.77	6.34(1.88)	1–9	0.85
Turkey	2.96(1.7)	1–8.5	0.80	3.22(1.76)	1–9	0.67	4.67(2.11)	1–9	0.87
Uganda	1.98(2.01)	1–9	0.90	2.10(2.05)	1–9	0.88	2.85(2.64)	1–9	0.90
USA	2.77(1.59)	1–9	0.83	2.08(1.43)	1–9	0.82	3.99(2.00)	1–9	0.87

Note. α = Cronbach’s alpha.

**Table 4:** Correlations among variables.

	1	2	3	4	5	6	7	8	9	M	SD
1.Biological Sex	1									==	==
2.Age	−0.02	1								20.35	3.63
3. Machiavellianism	0.14 **	−0.02	1							3.39	2.00
4. Psychopathy	0.18 **	0.04 **	0.58 **	1						2.85	1.76
5. Narcissism	0.08 **	0.05 **	0.55 **	0.39 **	1					4.42	2.19
6. Own sext	0.01	0.05 **	0.17 **	0.11 **	0.11 **	1				1.23	0.44
7.Risky sexting ^a^	0.08 **	0.05 **	0.28 **	0.22 **	0.15 **	0.55 **	1			1.28	0.51
8. Non-consensual sexting ^b^	0.11 **	−0.05 **	0.12 **	0.16 **	0.05 **	0.65 **	0.51 **	1		1.08	0.33
9. Sexting under pressure ^c^	0.09 **	−0.02	0.17 **	0.19 **	0.10 **	0.40 **	0.53 **	0.52 **	1	1.14	0.45

Note 1: ** *p* < 0.01. Biological sex was coded as 0 = girls and 1 = boys. ^a^ Correlations for risky sexting were run on a subsample of 5788 participants; ^b^ Correlations for non-consensual sexting were run on a subsample of 4983 participants; ^c^ Correlations for sexting under pressure were run on a subsample of 4704 participants.

**Table 5:** Sharing own sexts: parameter estimates of fixed effects.

			95% Confidence Interval			
	B	SE	Lower	Upper	*t*	*p*
(Intercept)	4.85	0.067	4.72	4.98	72.23	<0.001
Biological sex	−0.08	0.05	−0.17	0.014	−1.66	0.10
Age	0.03	0.01	0.011	0.04	3.90	<0.001
Machiavellianism	0.13	0.02	0.10	0.17	8.39	<0.001
Psychopathy	0.02	0.02	−0.01	0.06	1.42	0.16
Narcissism	0.03	0.01	0.002	0.05	2.14	0.03
Sex * Machiavellianism	−0.04	0.03	−0.10	0.02	−1.30	0.20
Sex * Psychopathy	0.06	0.03	−0.00	0.12	1.93	0.053
Sex * Narcissism	0.00	0.02	−0.05	0.05	0.001	0.99
Age * Machiavellianism	−0.01	0.004	−0.01	0.002	−1.42	0.16
Age * Psychopathy	0.01	0.004	0.001	0.02	2.26	0.02
Age * Narcissism	0.001	0.003	−0.005	0.008	0.34	0.74

Note. Unstandardized coefficients are reported. Biological sex was coded as 0 = girls and 1 = boys. Number of observations = 6093.

**Table 6:** Sharing own sexts: estimates of random component.

Groups		SD	Variance	ICC
Country	Intercept	0.206	0.0425	0.0140
	Residual	1.729	2.9910	

Note. ICC = intra class correlation.

**Table 7:** Risky sexting: parameter estimates of fixed effects.

			95% Confidence Interval			
	B	SE	Lower	Upper	*t*	*p*
(Intercept)	1.26	0.03	1.21	1.31	48.36	<0.001
Sex	0.03	0.01	0.001	0.06	2.04	0.04
Age	0.01	0.002	0.005	0.01	4.27	<0.001
Machiavellianism	0.06	0.005	0.05	0.07	12.49	<0.001
Psychopathy	0.03	0.005	0.02	0.04	5.46	<0.001
Narcissism	−0.00	0.004	−0.01	0.01	−0.10	0.92
Sex * Machiavellianism	0.004	0.009	−0.01	0.02	0.43	0.67
Sex * Psychopathy	0.014	0.009	−0.005	0.03	1.47	0.14
Sex * Narcissism	0.002	0.007	−0.01	0.02	0.27	0.79
Age * Machiavellianism	0.00	0.001	−0.002	0.003	0.65	0.52
Age * Psychopathy	0.002	0.001	−0.00	0.005	1.89	0.06
Age * Narcissism	−0.00	0.00	−0.002	0.002	−0.14	0.89

Note. Unstandardized coefficients are reported. Biological sex was coded as 0 = girls and 1 = boys. Number of observations = 5788. Malaysia was not included in the model because the measure of risky sexting was not available.

**Table 8:** Risky sexting: estimates of random component.

Groups		SD	Variance	ICC
Country	Intercept	0.079	0.00624	0.0246
	Residual	0.497	0.24698	

Note. ICC = intra class correlation.

**Table 9:** Non-consensual sexting: parameter estimates of fixed effects.

			95% Confidence Interval			
	B	SE	Lower	Upper	*t*	*p*
(Intercept)	8.78	0.18	8.43	9.12	49.63	<0.001
Sex	0.42	0.08	0.26	0.59	5.03	<0.001
Age	−0.03	0.01	−0.06	−0.01	−2.87	0.004
Machiavellianism	0.09	0.03	0.03	0.14	3.10	0.002
Psychopathy	0.21	0.03	0.15	0.27	7.36	<0.001
Narcissism	−0.03	0.02	−0.07	0.01	−1.47	0.14
Sex * Machiavellianism	−0.02	0.05	−0.13	0.08	−0.44	0.66
Sex * Psychopathy	0.18	0.06	0.07	0.29	3.28	0.001
Sex * Narcissism	−0.03	0.04	−0.11	0.06	−0.64	0.52
Age * Machiavellianism	−0.01	0.01	−0.03	0.001	−1.80	0.07
Age * Psychopathy	−0.00	0.01	−0.02	0.01	−0.12	0.90
Age * Narcissism	0.01	0.01	0.001	0.02	2.16	0.03

Note. Unstandardized coefficients are reported. Biological sex was coded as 0 = girls and 1 = boys. Number of observations = 4983. Analysis for non-consensual sexting was run only on the subsample of participants who reported to have or have had a dating partner.

**Table 10:** Non-consensual sexting: estimates of random component.

Groups		SD	Variance	ICC
Country	Intercept	0.57	0.319	0.0418
	Residual	2.71	7.321	

Note. ICC = intra class correlation.

**Table 11:** Sexting under pressure: parameter estimates of fixed effects.

			95% Confidence Interval			
	B	SE	Lower	Upper	*t*	*p*
(Intercept)	1.15	0.04	1.08	1.22	31.65	<0.001
Sex	0.08	0.01	0.05	0.11	5.34	<0.001
Age	−0.004	0.002	−0.01	−0.00	−2.22	0.03
MACHIAVELLIANISM	0.03	0.005	0.02	0.03	5.26	<0.001
PSYCHOPATHY	0.03	0.005	0.02	0.04	5.21	<0.001
NARCISSISM	0-00	0.004	−0.01	0.01	0.15	0.88
Sex * MACHIAVELLIANISM	−0.01	0.01	−0.03	0.01	−1.38	0.17
Sex * PSYCHOPATHY	0.04	0.01	0.02	0.06	4.41	<0.001
Sex * NARCISSISM	−0.003	0.01	−0.02	0.01	−0.41	0.69
Age * MACHIAVELLIANISM	−0.004	0.001	−0.01	−0.001	−3.04	0.002
Age * PSYCHOPATHY	0-00	0.001	−0.002	0.003	0.37	0.71
Age * NARCISSISM	0.002	0.001	−0.00	0.004	1.80	0.07

Note. Unstandardized coefficients are reported. Biological sex was coded as 0 = girls and 1 = boys. Number of observations = 4704. Analysis for sexting under pressure was run only on the subsample of participants who reported to have or have had a dating partner. Moreover, Malaysia was not included in the model because the measure of sexting under pressure was not available.

**Table 12:** Sexting under pressure: estimates of random component.

Groups		SD	Variance	ICC
Country	Intercept	0.112	0.0125	0.0546
	Residual	0.465	0.2159	

Note. ICC = intra class correlation.

## Data Availability

Data are available under request to the first author.

## Appendix A. Items of Each Dimensions of Sexting Behaviors Questionnaire (SBQ)

INSTRUCTIONS. Sexting has been defined as sending or receiving sexually suggestive or provocative messages/ photos / videos via mobile phone and/or Facebook or other Internet social networking site. Please answer the questions about your sexting behaviors during the last year using the following response scale:

1 = never; 2 = seldom; 3 = 2/3 times a month; 4 = 2/3 times a week; 5 = almost daily

**Table A1:** Items of Each Dimensions of Sexting Behaviors Questionnaire (SBQ).

Sexting Dimensions	SBQ Items
Own Sexts	How often have you privately sent provocative or sexually suggestive photos about yourself? How often have you publicly posted provocative or sexually suggestive photos about yourself on the web or social network sites? How often have you privately sent provocative or sexually suggestive videos about yourself? How often have you publicly posted provocative or sexually suggestive videos about yourself on the web or social network sites?
Risky sexting	How often have you sext when you were drinking alcohol? How often have you sext when you were smoking marijuana? How often have you sext when you were doing other drugs? How often have you sext with strangers or someone you know only from online?
Non-Consensual Sexting	How often have you privately sent provocative or sexually suggestive photos about your partner **without his/her consent**? How often have you privately sent provocative or sexually suggestive photos about someone you know **without his/her consent**? How often have you privately sent provocative or sexually suggestive videos about your partner **without his/her consent**? How often have you privately sent provocative or sexually suggestive videos about someone you know **without his/her consent**? How often have you publicly posted on the web or social network sites provocative or sexually suggestive photos about your partner **without his/her consent**? How often have you publicly posted on the web or social network sites provocative or sexually suggestive photos about someone you know **without his/her consent**? How often have you publicly posted on the web or social network sites provocative or sexually suggestive videos about your partner **without his/her consent**? How often have you publicly posted on the web or social network sites provocative or sexually suggestive videos about someone you know **without his/her consent**?
Sexting under Pressure	How often have you sext because your partner forced you? How often have you sext because your friends forced you?
